# Killer Cell Immunoglobulin-like Receptors (KIR) and Human Leucocyte Antigen C (HLA-C) Increase the Risk of Long-Term Chronic Liver Graft Rejection

**DOI:** 10.3390/ijms232012155

**Published:** 2022-10-12

**Authors:** Isabel Legaz, Jose Miguel Bolarín, Jose Antonio Campillo, María R. Moya-Quiles, Manuel Miras, Manuel Muro, Alfredo Minguela, María R. Álvarez-López

**Affiliations:** 1 Department of Legal and Forensic Medicine, Biomedical Research Institute (IMIB), Regional Campus of International Excellence “Campus Mare Nostrum”, Faculty of Medicine, University of Murcia, 30120 Murcia, Spain; 2 Immunology Service, Instituto Murciano de Investigación biosanitaria (IMIB), Centro de Investigación Biomédica en Red de Enfermedades Hepáticas y Digestivas (CIBERehd), Hospital Clínico Universitario Virgen de la Arrixaca (HCUVA), 30120 Murcia, Spain; 3 Digestive Medicine Service, Hospital Clínico Universitario Virgen de la Arrixaca (HCUVA), 30120 Murcia, Spain

**Keywords:** killer cell immunoglobulin-like receptors (KIRs), human leucocyte antigen (Hla), liver transplantation, chronic rejection, alcoholic cirrhosis, long-term graft survival

## Abstract

Chronic liver rejection (CR) represents a complex clinical situation because many patients do not respond to increased immunosuppression. Killer cell immunoglobulin-like receptors/Class I Human Leukocyte Antigens (KIR/HLA-I) interactions allow for predicting Natural Killer (NK) cell alloreactivity and influence the acute rejection of liver allograft. However, its meaning in CR liver graft remains controversial. KIR and HLA genotypes were studied in 513 liver transplants using sequence-specific oligonucleotides (PCR-SSO) methods. KIRs, human leucocyte antigen C (HLA-C) genotypes, KIR gene mismatches, and the KIR/HLA-ligand were analyzed and compared in overall transplants with CR (n = 35) and no-chronic rejection (NCR = 478). Activating KIR (aKIR) genes in recipients (rKIR2DS2^+^ and rKIR2DS3^+^) increased CR compared with NCR groups (*p* = 0.013 and *p* = 0.038). The inhibitory KIR (iKIR) genes in recipients rKIR2DL2^+^ significantly increased the CR rate compared with their absence (9.1% vs. 3.7%, *p* = 0.020). KIR2DL3 significantly increases CR (13.1% vs. 5.2%; *p* = 0.008). There was no influence on NCR. CR was observed in HLA-I mismatches (MM). The absence of donor (d) HLA-C2 ligand (dC2^−^) ligand increases CR concerning their presence (13.1% vs. 5.6%; *p* = 0.018). A significant increase of CR was observed in rKIR2DL3^+^/dC1^−^ (*p* = 0.015), rKIR2DS4/dC1^−^ (*p* = 0.014) and rKIR2DL3^+^/rKIR2DS4^+^/dC1^−^ (*p* = 0.006). Long-term patient survival was significantly lower in rKIR2DS1^+^rKIR2DS4^+^/dC1^−^ at 5–10 years post-transplant. This study shows the influence of rKIR/dHLA-C combinations and aKIR gene-gene mismatches in increasing CR and KIR2DS1^+^/C1-ligands and the influence of KIR2DS4^+^/C1-ligands in long-term graft survival.

## 1. Introduction

Chronic liver rejection (CR) represents a complex clinical situation because many patients do not respond to increased immunosuppression, which often leads to retransplantation or death [1,2]. Despite this, its incidence from the transplant is performed until the patient’s death or loss of the graft has decreased, and occurs in 3–17% of liver transplant recipients due to improved immunosuppression regimens in liver transplant recipients [3,4,5,6,7]. CR’s pathogenesis is multifactorial and is characterized by obliterative arteriopathy and ductopenia [4,8,9].

It has been demonstrated that adaptive and innate immunity through natural killer (NK) killer NK cells may be involved in immune responses in the liver [10,11]. The liver contains the most significant NK cell population [12,13]. These innate lymphocytes are crucial in screening for infection and liver pathology, and their nature and functions have been a focus of recent interest [14,15]. NK cells express a balance of activating and inhibitory receptors and allow alloactivation to be defined, among others, by different models of KIR/KIR-ligand interactions. Inhibitory and activating killer cell immunoglobulin-like receptor (iKIR and aKIR) mismatching may play a role in graft-versus-host-disease (GVHD) and liver survival [16,17]. In organ transplantation, some studies have suggested the role of KIR/HLA-C-ligands in liver and kidney transplants and their consequences for acute rejection and short-term liver allograft injury [17,18,19,20,21,22]. A previous study revealed that NK cells could perform two functions after solid organ donation. These cells can improve tolerance in immunocompromised environments while also exacerbating the rejection process by amplifying the immune responses associated with rejection [23,24]. I NK cells have been found to have a significant role in the pathogenesis of both acute and chronic antibody-mediated rejection (ABMR) and T cell-mediated rejection (TCMR) among all innate immune cells linked with allograft rejection (TCMR) [25]. Higher NK cell cytotoxicity was found to be associated with kidney allograft rejection, as evidenced by increased CD107a expression and interferons (IFN-)- production in patients with acute and chronic renal transplant rejection compared to those with well-functioning grafts [22].

KIRs are glycoproteins expressed on NK-cells and certain T lymphocyte subsets [26,27]. KIR receptors contribute to fine-tuned NK cell activity regulation by binding their extracellular domains to restricted residues on HLA-I molecules [28]. Long or short intracytoplasmic KIR tails transmit inhibitory (iKIR) or activating (aKIR) signals inside the cell [29,30]. Eight KIR genes (KIR2DL1-3 and KIR2DL5 and KIR3DL1-3) encode iKIR receptors, and six aKIR receptors (KIR3DS1 and KIR2DS1-5). KIR2DL4, encode a receptor with both functions [31,32].

KIR genes exhibit extensive haplotypic variation in gene number [33] and allelic polymorphism in all human populations [34,35]. Within iKIRs, KIR2DL1 recognizes a lysine at the 80 positions on the α-helix of alleles belonging to the HLA-C2 allotype, while KIR2DL2/3 identifies asparagines at the same position on HLA-C1 alleles. The binding of aKIRs to HLA-C-ligands has only been documented for KIR2DS1 and KIR2DS4, with the former recognizing C2 ligands [36,37] and the latter, both C2 and a limited number of C1 ligands [38], while ligands for other aKIRs remain unknown.

KIR/HLA-C genotyping in both the graft and the recipient’s liver could help the pathologist predict rejection responses and estimate survival to the point where clinical steps can be taken to save the graft and increase the quality and life expectancy of the transplanted patient. The impact of KIR/HLA-I on the establishment of CR and survival was examined in this study, focusing on recipient KIRs (rKIR) and donor HLA (dHLA) class I ligands.

## 2. Results

### 2.1. Transplant Clinical Endpoints

In this cohort, the most frequent transplant indications were alcoholic cirrhosis (32.9%) and chronic viral hepatitis (21.1%); men were more represented than women (74.5% and 25.5%); and the recipients of HCV infection represented 30.2% while that of the HBV was of 9.9% (Table 1). CR occurred in 6.8% of all patients (n = 35 of 513). The patients included in this study developed CR after 3.34 ± 0.7 years (mean ± SEM) post liver transplant.

Although CR was not associated with the recipient age, gender, transplant indications, or viral pre-infections, a statistically significant reduction of mean donor age was found in the group of patients suffering from CR concerning those of the NCR group (44.09 ± 3.61 versus 51.8 ± 0.9 years; *p* = 0.026).

### 2.2. Impact of KIR Genes and KIR Genotypes in Chronic Rejection

The impact of KIRs and HLA-I-ligands on long-term liver graft outcomes was analyzed in overall transplants and the CR and NCR groups. As shown in Table 2, the KIR gene distribution in patients and healthy donors was similar, and in no case were significant differences found, except for KIR2DS5, which was significantly up-represented in liver recipients for healthy donors (33.9% vs. 27.9%; *p* = 0.043). The effect of each KIR gene on CR was also evaluated.

KIR2DL2 significantly increased CR for NCR patients (*p* = 0.020). Within aKIRs, KIR2DS2 is strongly associated with KIR2DL2, and KIR2DS3 showed a significant increase in patients developing CR episodes to compared to NCR (*p* = 0.013 and *p* = 0.038, respectively. All these associations were confirmed using multivariable logistic regression analysis.

**Figure 1:**
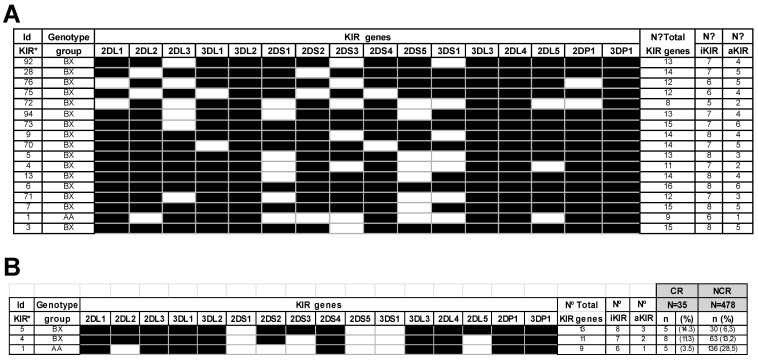
KIR genotypes profiles observed in patients. (**A**) Most frequent KIR genotypes profiles observed in CR patients (**B**) KIR genotypes profiles observed in patients with (CR) and without CR (NCR). The first row represents the international genotype classification (ID) of the KIR genotypes most represented in both populations. The second-row shows haplotype groups found in both populations. The third row represents each KIR analyzer’s presence (dark boxes) and absence (white boxes). The fourth, fifth, and sixth rows show each genotype’s total number of iKIR and aKIR genes. The seventh row represents patients’ genotype frequency (CR and NCR). N, number of individuals; KIR, Killer-cell immunoglobulin-like receptors; iKIR, Inhibitory killer-cell immunoglobulin-like receptors; aKIR, Activatory killer-cell immunoglobulin-like receptors.

On the other hand, the genotype KIR distribution was also examined (Figure 1A). This exam showed that genotype frequencies in healthy donors of our series were similar to those of a healthy Caucasoid population [39]. In CR patients, 17 different KIR genotypes of those previously reported in the Allele Frequencies website KIR database [39] were found, with 14 out of 17 represented only once or twice. However, no relation was found between the total number of KIR, iKIR, or aKIR genes and CR. Figure 1B shows that the most frequent KIR genotypes in CR patients were ID5-BX, ID4-BX, and ID1-AA. Interestingly, these genotype frequencies were inverted in the NCR group. Patients bearing the ID1-AA genotype (28.5%) showed a lower risk of suffering from CR.

### 2.3. The iKIR Gene Mismatching Increases the Chronic Rejection Rate

According to the KIR gene-gene model, the effect of KIR gene mismatching (MM) between recipient-donor pairs was also analyzed (Table 3), comparing liver transplants performed with 0 KIR gene mismatches (0 MM) with those done with one or more mismatches (≥1 MM). This analysis revealed a significant increase in the CR rate of transplants performed with ≥1 MM in iKIR genes) concerning those with 0 MM (8.1% and 2.5%, respectively; P1 = 0.037), a correlation confirmed by the multivariable analysis. In contrast, no significant association was found when mismatched overall KIR or aKIR genes were considered. Discrepancies in individual iKIR genes were also explored, but no effect was observed on CR, except for KIR2DL3. Thus, the CR rate was also significantly increased in transplants with ≥1 MM KIR2DL3 for those with 0 MM (respectively 13.1% vs. 5.2%; *p* = 0.008).

### 2.4. Impact of HLA-I Allele, HLA-C Allotype, and HLA-C Genotypes on Liver Allograft Chronic Rejection

Second, the possible influence on liver allograft CR of the HLA-A, -B, and -C allele mismatching between recipient-donor pairs (Table 4) and that of the HLA-C allotypes and genotypes of recipients and donors (Table 5) was also examined.

In no case was the CR development associated with the HLA-A, -B, and -C allelic incompatibility between recipient- donor pairs or with the HLA-C allotype of the recipient. However, the CR rate significantly increased compared to the C1-ligand absence or presence in donors (13.1% vs. 5.6%; *p* = 0.018), or donors bearing a C2/C2 homozygous genotype with those having any of the other two genotypes (*X^2^* = 0.043; *p* = 0.018

Additionally, following the ligand-ligand model, comparisons between the different combinations of recipient-donor pairs HLA-C genotypes revealed that despite the reduced number of cases developing CR in these groups, a progressive upward trend in CR could be observed when livers from C2C2 donors were respectively allocated in patients with C2C2 (5.6%) < C1C2 (13.9%) < C1C1 (16.7%) genotypes (Table 6).

### 2.5. Combinations between Recipients Bearing Both KIR2DL3 and KIR2DS4 and Liver Donors Having C2-Ligands Predispose to a Higher Chronic Rejection Risk

According to the receptor-ligand model, the potential NK-alloreactivity was investigated in the recipient versus donor direction (R→D) considering combinations of both iKIRs and aKIRs of recipients with their documented HLA-ligands present in donors. As KIR2DL1 was present in most individuals, patients with this KIR gene were categorized as KIR2DL1^+^/S1^-^ and KIR2DL1^+^/S1^+^ to better understand their inhibitory effect or activating capabilities.

Thus, the CR frequency was first independently evaluated in patients with inhibitory KIR2DL1^+^/S1^-^, KIR2DL2^+^/S2^+^, KIR2DL3^+^ or KIR3DL1^+^ and in those with activating KIR2DL1^+^/KIR2DS1^+^ and KIR2DS4^+^ genes who received livers from donors with any of the documented specific HLA-I ligands for each considered KIR.

As summarised in Table 7, within iKIRs-C-ligand possible combinations, the CR rate was notably higher and borderline to the significance in transplants done between KIR2DL2^+^/S2^+^ recipients and donors lacking C1-ligands (C2/C2 homozygous donors) compared with transplants in which donors were positives for C1-ligands (16.7% vs. 7.7%; *p* = 0.057).

An apparent CR increase was seen when transplants were performed between KIR2DL3^+^ recipients and donors lacking C1-ligand compared with those done between KIR2DL3^+^ recipients and donors possessing the C1-ligand (13.2% and 4.8%; *p* = 0.015), an association that was confirmed and improved when the multivariable analysis was applied (*p* = 0.004). No other iKIR/ligands combinations, including that of the KIR3DL1 with its Bw4-ligands, could be associated with significant changes in CR.

Regarding the aKIRs/HLA-C ligands, the CR was significantly increased in transplants combining KIR2DS4^+^ recipients and donors who lacked C1-ligands compared with those performed in KIR2DS4^+^ recipients having donors with C1-ligands (13.4% and 5.4%; *p* = 0.014). The remaining aKIR/HLA-C ligand combinations were not associated with the CR development.

Given the increase of CR detected transplants done in KIR2DL3^+^ and KIR2DS4^+^ patients receiving a liver from donors who wholly lacked C1-ligands, we also assessed the possibility of a synergic effect of these two KIRs (Table 7). This test revealed an increase in CR rate and statistical significance (13.5% vs. 4.5; *p* = 0.006) when patients possessing both KIR in their genome received allografts from donors in whom C1-ligands were absent. Results were also confirmed by using multivariable logistic regression analysis (*p* = 0.003).

**Figure 2:**
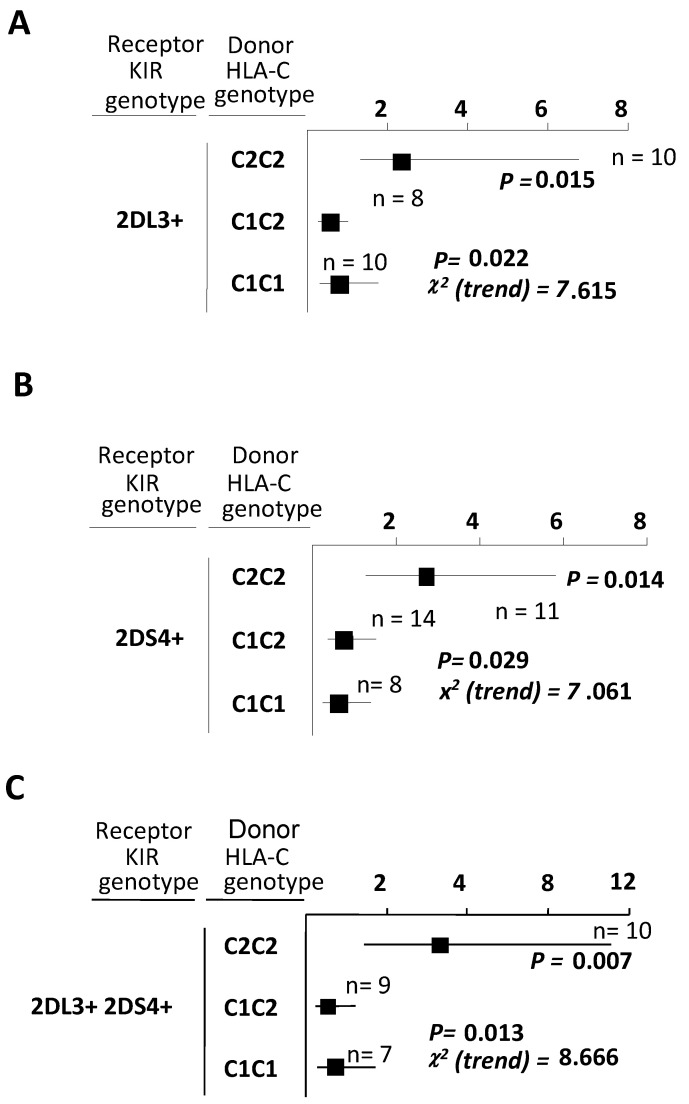
The cumulative effect on chronic rejection in KIR2DL3^+^, KIR2DS4^+,^ and KIR2L3^+^/KIR2DS4^+^ recipients depends on donor C2 ligands dose. (**A**,**B**) Illustrates Odds Ratios and the 95% confidence intervals (CI) for CR incidence in KIR2DL3 and KIR2DS4 patients, respectively, combined with donors having two C2-ligands (C2C2), one C2-ligand (C1C2), or lacking C2-ligands (C1C1 donors). Two-by-two contingency tables and the Chi-square test were used to determine the results, and statistically significant differences were found (χ^2^ = 7.615; *p* = 0.022 and χ^2^ = 7.061; *p* = 0.029, respectively). (**C**) Represents variations in OR and the 95% CI for CR in patients bearing KIR2DL3^+^ and KIR2DS4^+^ genes in their repertoires, with dependence on C2-ligand dose and statistically significant differences observed (χ^2^ = 8.666; *p* = 0.013). n, the number of patients included in each series, is indicated.

Finally, the analysis of the possible impact of donors’ C1 or C2-ligand doses on the allograft response of KIR2DL3^+^ or KIR2DS4^+^ recipients (Figure 2) showed that CR independently and significantly increased in both types of patients (χ^2^ = 7.615; *p* = 0.022 for KIR2DL3^+^, and χ^2^ = 7.061; *p* = 0.029 for KIR2DS4^+^ recipients), but only regarding the presence of double donor C2-ligand doses (Figure 2A and Figure 2B; *p* = 0.015 and *p* = 0.014 respectively). Furthermore, when examining all responses of recipients possessing both KIR2DL3 and KIR2DS4 against donors belonging to the different allotype groups (Figure 2C), a synergic effect on CR (χ^2^ = 8.666; *p* = 0.013) was again detected.

### 2.6. KIR/HLA-I-Ligand Combinations and Long-Term Graft Outcome

**Figure 3:**
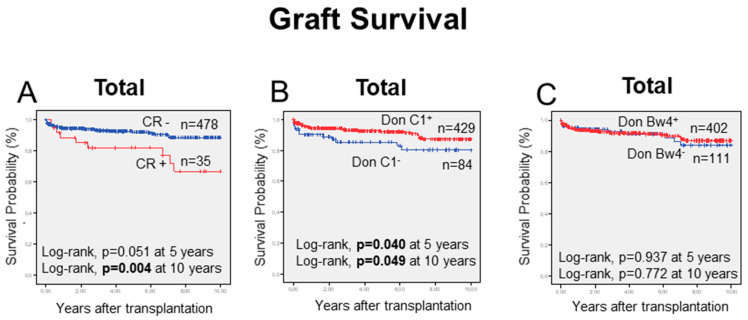
Kaplan-Meier graft survival curves following liver transplantation. (**A**) Description of the influence of chronic graft rejection (red line) in decreased graft survival 5 and 10 years after transplant. (**B**) The presence (red line) of C1-ligand in donors increased graft survival 5 and 10 years after transplant. (**C**) Illustration of the lack of influence of the Bw4-ligand in donors on graft survival 5 and 10 years after transplant.

On the other hand, in the overall transplants cohort, the long-term graft outcome was investigated, first drawing attention to the probability of allograft survival in patients showing CR episodes at 5 and 10 years as well as those depending on the HLA-C or -HLA-Bw4 ligands carried by the donor (Figure 3).

The allograft survival decreased in overall patients suffering from CR to those without CR episodes (Figure 3A). The observed differences were borderline significant at five and significant at ten years (respectively, *p* = 0.051 and *p* = 0.004). In another way (Figure 3B), the probability of graft survival significantly increased, at 5 and 10 years, in transplants having donors with HLA-C1-ligands for those in which the HLA-C1-ligand were absent donors (*p* = 0.040 and *p* = 0.049, respectively for 5 and 10 years). In contrast, no differences were found when the effect of donor bearing or not HLA-Bw4 ligands were examined (Figure 3C).

**Figure 4:**
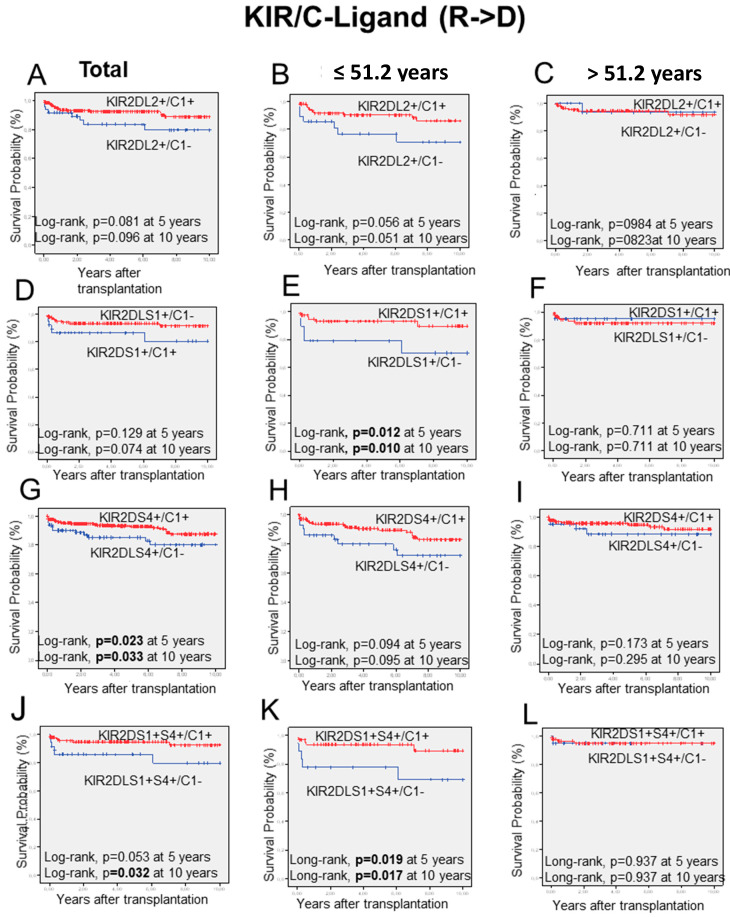
Kaplan-Meier graft survival curves following liver transplantation. This figure summarizes long-term graft survival for the presence or absence of KIR2DL2 (**A**–**C**), KIR2DS1 (**D**–**F**), and KIR2DS4 (**G**–**I**) in combinations with donors having C1-ligand (red lines) or lacking C1 (C2/C2 donors, blue lines), KIR2DS1 with KIR2DS4 (**J**–**L**) with donors having C1-ligand (red lines) or lacking C1 in the R→D direction in the three groups established. Illustration of the KIR2DS1/S4-C-ligand combinations, showing that donors with <52.1 years who received grafts from donors lacking C1-ligands (C2/C2 donors) exhibit the most significant fall in long-term graft survival, both at five years and ten years (Log-rank, *p* = 0.019, and *p* = 0.017).

In a second step, and once the Bw4-ligands effect was discarded, the influence of the KIRs/HLA-C pairs in long-term graft survival was tested following the *receptor-ligand model* in the recipient versus graft direction (R→D), but only for KIRs with documented abilities to bind some HLA-C alleles from the C1 and C2 allotypes (Figure 4).

Given the observed differences in CR associated with the donor age (*p* = 0.026), the impact of the KIR/HLA-C combinations on long-term liver graft outcome was evaluated in overall transplants and the two established age groups according to donor mean age (51.2 ± 0.9; mean ± SEM) years) given the observed differences in CR associated with the donor age.

Thus, among iKIR/HLA-C combinations of KIR2DL2^+^/C1^+^and KIR2DL2^+^/C1^−^ (Figure 4A–C) there were no significant differences in the long-term probability of graft survival of the total transplants cohort nor the group with donor upper 51.2 years, while in this of the younger donors (lower than 51.2 years) these differences were higher and borderline to the significance (*p* = 0.056 at five years and *p* = 0.051 at ten years; Figure 4B)

Furthermore, aKIR/HLA-C comparisons between KIR2DS1^+^/C1^+^ and KIR2DS1^+^/C1^−^ combinations did not influence overall transplants or those with older donors, whereas in transplants having younger donors, differences between KIR2DS1^+^/C1^+^ and KIR2DS1^+^/C1^−^ combinations (Figure 4D–F), there were again significant differences at 5 and 10 years after transplantation (*p* = 0.012 and *p* = 0.010 at 5 and 10 years, respectively).

Moreover, comparisons for KIR2DS4^+^/C1^+^ and KIR2DS4^+^/C1^-^ showed that long-term graft survival decreased when patients bearing KIR2DS4^+^ were combined with donors lacking C1-ligands, but differences were only significant when the total transplants cohort was considered (Figure 4G; *p* = 0.023 and *p* = 0.033 at 5 and 10 years), whereas, in any of the age donor groups, these differences did not reach significance (Figure 4H,I).

However, as it is shown in (Figure 4K), the log-rank highest difference in graft survival was detected when recipients who bear both KIR2DS1 and KIR2DS4 received livers from donors possessing a C1-ligand (C1/C1 or C1/C2 donors) were compared with those having donors who lack the C1-ligands (C2/C2 donors, Log-rank, *p* = 0.019 and *p* = 0.017 at 5 and 10 years, respectively).

## 3. Discussion

In this retrospective study, we have analyzed in a cohort of patients undergoing liver transplants the variations in KIR/HLA-C gene content in recipient-donor and its possible relationship with CR development and graft survival for the first time.

The incidence of CR in liver transplants is reported as between 3% and 17%, much lower than that observed for AR episodes and other solid organs such as the heart (25–60%), pancreas (20–40%) or kidney (30–70%) [7]. In our study, CR is represented at 6.8%, a usual range in the liver. Although the rate of CR has been declining over the past two decades, some current circumstances justify continued vigilance over this complication [7]. Risk CR factors include the donor’s advanced age, the autoimmune nature of the pretransplant disease, and the reduction or voluntary suspension of immunosuppression [40,41,42,43]. Thus, donor selection in liver transplantation is one of the most critical factors contributing to transplant success [44,45]. An age limit of 65 years for liver donation is reasonable [46]. The average age of liver donors has been increasing in recent years, reaching 61.3 years for adults [46]. In our study, the mean age of the donor (51.2 ± 0.9 years) is within the recommended values. However, our data show how younger donors (44.09 ± 3.61 years) are statistically associated with a higher CR development frequency in the NCR group (51.8 ± 0.9 years). On the other hand, other studies observed that donor age does not affect patient survival. At the same time, an increased rate of delayed non-function of liver grafts was procured from older donors [47].

Several publications relate KIR/HLA-I receptors to the effects of AR and patient survival in different organ transplants. However, no studies have examined the influence of CR on liver grafts [17,48,49].

This study analyzed the frequencies of KIR genes in donor-patient pairs. A high frequency of the KIR2DS5 gene was observed for healthy controls but not related to CR development. In contrast, a higher and more significant frequency of KIR2DL2/S2 and KIR2DS3 was observed in CR patients. However, other studies of AR showed no association with the presence or absence of individual KIR genes in liver receptors [17,49], but further studies in AR in kidney grafts observed a protective effect of the KIR2DS5 gene and association of the KIR2DS4 gene [50,51,52].

KIR genotypes were also analyzed in patients. It was observed that those patients with the ID1-AA genotype (with only one aKIRs) showed a low risk of suffering from CR, while the ID5-BX genotype (with three aKIRs) was more represented in NCR patients. In any case, there is no statistically positive relationship between the number of aKIRs and CR development. Similarly, other studies observed that the number of activating or inhibitory KIR genes in the recipient genotype was not associated with liver allograft AR or graft survival [17,49].

Functional mechanisms explaining the beneficial effects of KIR-genotype matching are as yet hypothetical. KIR-matching may increase the degree of genetic similarity between recipient and donor by increasing the number of genes inherited, resulting in a decrease in GVHD and graft-versus-leukemia (GVL) effects. The impact of donor-recipient KIR gene-gene matching on transplant outcomes is inconclusive. However, various studies suggest the MM’s implication in resolving allogeneic hematopoietic stem cell transplantation [48,53,54].

Our data show that in liver transplantation MM of iKIR seems to favor CR and, more specifically, the KIR2DL3 MM. However, any effect was observed in aKIRs MMs about CR.

Other similar studies in liver transplants, however, found a progressive and significant increase in AR with the number of KIR gene discrepancies compared to transplants fully matched (0 MM) with the group having one or more MMs (≥1 MM) in total KIR genes, with the highest significance observed when comparing the 0 MM and ≥1 MM groups for aKIRs.

In contrast, for iKIRs, AR was not significantly different [17]. All these data seem to indicate the existence of a chronology in the MM of KIR genes that seems to influence the mechanisms of liver graft rejection, influencing the discrepancy of aKIR in the short term and the discrepancy between iKIR in the long term.

On the other hand, any CR associations were found with HLA-A, -B, and -C allele mismatches, and similar observations were found in AR in liver transplants [17]. In contrast, the analysis of the HLA-C genotype of recipient and donor pair showed a significant influence of donor C2C2 in CR development, showing the highest CR rate (13.1%), only exceeded when the recipient was of the genotype C2C2 (16.7%). Other studies observed that AR was significantly associated with the HLA-C genotype of recipients and differently associated with C1- and C2-homozygous recipients who received allografts from C1/C2 donors [17,55]. On the other hand, Lee H et al. [49] demonstrated that patients who received liver transplants from donors with the HLA-C2 allele had a higher risk of AR than those who received livers from donors with a homozygous HLA-C1 genotype.

Considering the receptor-ligand model, the results of this study confirm that the KIR2DL3^+^/C2-ligand combination significantly increases CR in our patients. Given the higher affinity of KIR2DL3 for the C1 ligand [56], it is not easy to reconcile why KIR2DL3/C2 combinations result in higher CR activation, similarly, but not significantly, to the KIR2DL2/C1-ligand combination. Similarly, another study reported that KIR2DL3 and KIR2DS1 in patients increased AR incidence in the presence of donor C2 ligands [17].

The lack of significant inhibition by KIR2DL2 and KIR2DL3 in our series could be explained by the smaller number of recipient-donor pairs carrying this allele and its ability to identify C1 and C2 ligands, or it could be attributable to reduced KIR2DL2/L3 expression [57,58]. Nonetheless, since KIR2DL2 is in linkage disequilibrium with its activating counterpart, and both genes are expressed together on NK cell surfaces [28,59,60], a subliminal counterbalance favored by the greater likelihood of KIR2DS2/C1-ligand interaction should not be discarded if the availability of the HLA-I ligand arises in the context of allogeneic pro-inflammatory microenvironment encompassing acute rejection increase.

**Figure 5:**
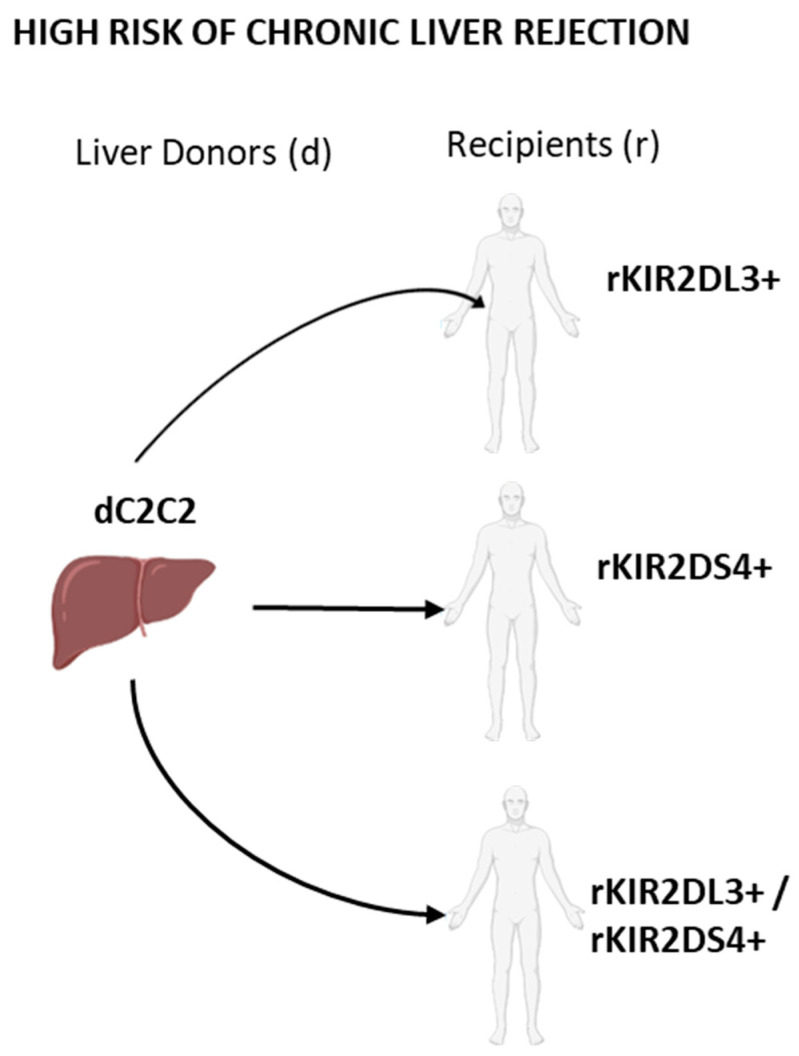
Representative scheme of the main combinations of KIR/HLA-c combinations with a high risk of chronic liver rejection. d, donor. r, recipient.

Concerning KIR2DS4, although its functional ligands have yet to be identified, this receptor might bind some HLA-I alleles [38,61], and other unknown ligands, such as non-MHC class-I proteins [62]. However, whether these interactions may activate some specific functions in NK cells remains poorly understood. However, in any case, our results showed a higher CR rate in KIR2DS4/C1- than in KIR2DS4/C1^+^ transplants. On the contrary, other studies did not observe any relationship between KIR2DS4^+^ and acute liver rejection [17]. This fact is also observed in patients with KIR2DL3^+^/S4^+^ in the absence of the C1 ligand (C2C2) in the liver graft (Figure 5).

It should be noted that both genes are present in the most abundant genotypes (Id 5, 4, and 1) in CR patients. On the other hand, a significantly higher risk of CR after kidney transplantation was observed when the recipient (r) and donor (d) pairs completely lacked the two functional rKIR-dHLA ligand combinations rKIR2DL1/dHLA-C2 and rKIR3DL1/dHLA-Bw4. This immunogenetic profile corresponds to low levels of NK cell inhibition [63]. However, our data do not show any association of rKIR2DL1/dHLA-C2 with CR, even though KIR3DL1 is present with KIR2DS4 in the most frequent CR genotypes.

**Figure 6:**
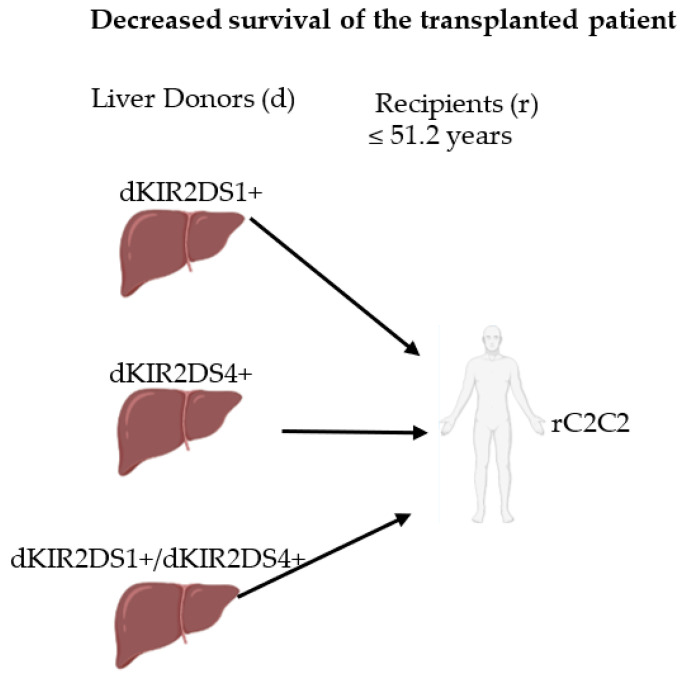
Representative scheme of the main KIR/HLA-C combinations with decreased survival of transplanted patients at 5 and 10 years. d, donor. r, recipient.

Finally, the influence of KIR/C-ligand on graft survival was also analyzed. A significant reduction in survival was observed when the donor lacked the C1-ligand. These same observations have also been observed for acute liver graft rejection [17,18,49]. The reduction in survival was more pronounced in dKIR2DS1^+^/rC1^−^ transplants and dKIR2DS4^+^/rC1^−^ transplant patients with both combinations at 5 and 10 years (Figure 6). It should be noted that a similar but not statistically significant effect is observed in transplants with dKIR2DL2^+^/rC1^−^. Similar data regarding survival in patients with acute liver graft rejection is observed with dKIR2DS1^+^/rC1^−^ and dKIR2DS4^+^/rC1^−^ transplants; however, these authors also observed the effect of dKIR2DL3^+^/rC1^−^ [17].

Our findings highlight the importance of KIR and KIR ligand-dependent alloreactivity in late liver allograft outcomes, suggesting that each KIR^+^ cell is “encouraged” to sense the missing ligand. Each aKIR^+^ cell binds putative ligands on allogeneic cells. Thus, increased cytotoxicity and activation of NK or T cells contribute to an inflammatory environment that favors short-term liver allograft damage. Furthermore, our findings demonstrate that categorizing transplants by KIR/HLA-I ligand pairings rather than entire cohorts allows for a more accurate assessment of long-term liver allograft damage because components that mediate contradicting effects are hidden in whole series analysis. Further investigation of allelic differences in KIR and HLA ligands, gene copy number variations and their expression levels, and the high morbidity-mortality rates of patients in the early post-transplantation period are all needed to confirm these findings. The findings of this study could help prevent CR and serve as a starting point for tailored therapy. The findings of this study could help prevent CR and, as a starting point for tailored therapy, better and more precise control of the patient by modulating immunotherapy.

## 4. Material and Methods

### 4.1. Patient Enrollment

A total of 513 successive liver transplants recruited from the Virgen de la Arrixaca Hospital in the Murcia Region (Spain) between 1990 and 2013 were analyzed retrospectively.

The inclusion criteria were: primary whole liver allograft without prior history of other organ transplants or rejections, ABO compatibility, and HIV-negativity. After excluding pediatrics and recipients losing liver grafts within the first week following transplantation, the final series comprised 513 transplants in which both patients and donors were Caucasian individuals.

The main characteristics of donors and patients with different liver transplant indications are summarized in Table 1. The mean age of both recipients (patient) and liver donors was similar [51.2 ± 0.9 and 53.0 ± 0.5 years (mean years ± SEM)], respectively. A total of 74.5% of the transplants were performed on men (n = 382) and 25.5% on women (n = 131). All patients gave informed consent to clinical information and follow-up data. The institutional ethical committee approved the study protocol according to the Declaration of Helsinki 2000.

### 4.2. Chronic Liver Rejection Diagnosis

The CR diagnosis was based on clinical, radiological, laboratory, and histopathology findings. Histological criteria for CR diagnosis included lymphocyte bile duct damage in 50% or more of the portal triads, with evidence of bile duct loss and hepatocanalicular cholestasis [64]. Patients were divided into two groups for data analysis: those with CR (n = 35) and those without CR or NCR (n = 478) (Table 1). The total follow-up CR time of the patients was ten years.

### 4.3. Viral Pre-Infection Diagnosis

Viral Hepatitis C (HCV), hepatitis B (HBV), and CMV pre-infections were analyzed. The presence of Hepatitis C Virus (HCV) was determined using a qualitative immunoassay (AxSYM HCV v 3.0, Abbott, Wiesbaden-Delkenheim, Germany) to detect the presence of anti-hepatitis C antibodies. The results were confirmed by immunoblotting technology (RIBA) or RT-PCR (REAL, C.E. Durviz, Valencia, Spain), following the manufacturer’s indications.

The HBV infection was determined by measuring the hepatitis B virus surface antigen (HBsAg) by a radioimmunological method (Sorin Biomedica, Perugia, Italy) and CMV infection, testing anti-CMV IgG antibodies by immunoassay (Liason^®^ CMV-IgG, DiaSorin, Saluggia, Italy). IgG antibodies at a level ≥0.6 IU/mL was used for the initial assignment of the CMV infection. A real-time PCR (LightCycler^®^ CMV-Quant-kit, Roche, Indianapolis, IN, USA) was used to corroborate the data of these positive instances.

### 4.4. Immunosuppressive Treatment

Immunosuppression consisted of standard triple-drug therapy with cyclosporine A or tacrolimus, methylprednisolone, and mycophenolate. Cyclosporine or tacrolimus was given on the first day following transplantation. The dose was adapted according to blood concentrations and clinical complications, such as renal function disorders and rejection episodes. Methylprednisolone was administered in the perioperative period at an initial dose of 1g, subsequently adjusted to 20 mg/day. Mycophenolate was initiated at a standard dose of 2 g/day and adjusted at 0.5 to 3 g/day according to leukocyte counts and digestive tolerance. Treatment of CR was based on immunosuppression intensification with high-dose methylprednisolone (one bolus of 500 mg for 3 days).

### 4.5. KIR and HLA Typing and Genotype Assignment

Both patients and donors were genotyped for KIR and HLA-I. To this end, genomic DNA was extracted using the QIAamp DNA Blood Midi Kit (QIAGEN, Hilden, Germany). KIR genotyping was performed using sequence-specific oligonucleotides (PCR-SSO) by Luminex^®^ technology (Tepnel Lifecodes, Stamford, CT, USA) and sequence-specific primers (PCR-SSP) according to the Vilches et al. protocol [65]. KIR and HLA genotypes were analyzed in all patients in this study at the time of DNA extraction prior to liver transplantation.

A total of 16 KIR genes were analyzed, nine of them inhibitory KIR (KIR2DL1-5, KIR3DL1-3), six activating (KIR2DS1-5, KIR3DS1), and two pseudogenes (KIR2DP1 and KIR3DP1), but KIR2DL5A and KIR2DL5B genes could not be distinguished. Both genes were investigated jointly because KIR2DL2 is tightly connected with KIR2DS2, and their personal effects could not be differentiated in our population [66]. KIR genotype profiles were categorized for further study using the Allele Frequencies KIR Database website [39]. Each genotype was named based on the worldwide genotype number (ID).

For KIR genotype assignment, the KIR gene content of individuals was used to infer the A and B KIR haplotypes and assign them to the AA, BB, and AB, genotypes [67]. Individuals carrying only KIR3DL3, 2DL3, 2DL1, 2DP1, 3DP1, 2DL4, 3DL1, 2DS4, and 3DL2 genes were considered to be homozygous for the A haplotype. Conversely, individuals who lacked KIR2DL1, 2DL3, 3DL1, or 2DS4 genes were carriers for two B KIR haplotype copies [68]. The remaining people were thought to be AB heterozygous or AB genotypes. These individuals possessed all the nine genes present in the A haplotype and one or more of the B haplotype genes (KIR2DL2, 2DL5, 2DS1, 2DS2, 2DS3, 2DS5, and 3DS1). KIR genotype BX was created by combining the AB and BB genotypes [68]. Each KIR gene was assigned to the centromeric or telomeric regions in the KIR gene complex [54,69].

HLA-I was genotyped using the Dynal RELI^TM^ SSO HLA-C Kit (Dynal Biotech ASA, Oslo, Norway) at a level of resolution necessary to distinguish the dimorphism at position 80 on the α1-helix. According to this dimorphism, HLA-C alleles were assigned to the two primary KIR ligand HLA-C allele groups: HLA-C1 and HLA-C2, and to the three different HLA-C genotypes C1C1, C1C2, and C2C2 [17,55,70].

### 4.6. KIR-Ligand Models and NK-Alloreactivity Prediction

The different models of KIR/HLA-ligand interactions proposed to infer the likelihood of NK-alloreactivity in HSCT [71] have been algorithms that were also applied to investigate the role of KIR and HLA-class-I ligands on potential NK-alloreactivity in liver transplantation, such as those previously reported for short-term liver allograft injury [17].

With a similar purpose, these three KIR-ligands models have been used in the present study to assess the potential effect of NK-alloreactivity on CR and long-term patient survival. Thus, the KIR gene-gene model was useful to test the disparity in KIR genes between recipients and donors when some KIR genes of the recipient was not included in the KIR genotype of donors (R^+^ D^−^) or when KIR genes present in the recipient were absent in the donor genotype. The HLA-I ligand-ligand or missing-self model, defined for KIR-ligand mismatching between donors and recipients as well as the receptor-ligand or missing-ligand model, was suitable for KIR and HLA-ligand combinations when donors lacking ligands for inhibitory KIR receptors present in the recipient genotype were taken into account to evaluate NK-alloreactivity in the long-term recipient-versus-donor direction (R→D). In this analysis, the analyzed 11KIR genes contain five inhibitory KIR genes (2DL1-3, 2DL5, 3DL1) and six activators (2DS1-S5 and 3DS1).

### 4.7. Statistical Analysis

The demographic information and findings were entered into a database (Microsoft Access 2.0; Microsoft Corporation, Seattle, WA, USA) and analyzed with SPSS 15.0. (SPSS Software Inc., Chicago, IL, USA). Potential NK-cell alloreactivity was evaluated according to the Hematopoietic Stem Cell Transplant (HSCT) models and our previous study [17]. All results were expressed as mean ± standard error of the mean (SEM) or as a percentage. Pearson’s χ^2^ and the two-tailed Fisher’s exact tests were used to compare categorized variables between groups, and a two-sided Student *T*-test and the nonparametric Mann-Whitney test were used to compare mean values. Positive relationships were confirmed using multivariable logistic regression analysis.

Correlations between CR and the presence or absence of individual KIR genes or KIR genotypes, patients’ and donors’ HLA-I-allotypes, and KIR/HLA-ligand combinations were also analyzed. The odds ratio (OR) and 95% confidence interval (CI) were also calculated, and *p*-values below 0.05 were significant. The multivariable logistic regression analysis was done considering the following variables: recipient’s age, sex, HCV pre-infection, KIR genotypes, donor’s age, and HLA-C allotype groups, as well as the KIR/C-ligands match or mismatching between recipients and donors.

The Kaplan-Meier method and Log-rank test compare differences in long-term graft survival at 5–10 years. The initial time of all survivals analyzed in our study corresponds to the date of liver transplantation of each patient.

The multivariable Cox proportional hazard model for the following variables: HLA mismatches, donor HLA-C allotypes, age, sex, and HCV pre-infection was also considered to confirm differences in graft survival.

## Figures and Tables

**Table 1:** Baseline demographic and clinical characteristics of liver donors and patients.

	n (%)	Age (Mean ± SEM)
**Total of Recipients, n (%)**	513	53.0 ± 0.5
CR group	35 (6.8)	49.9 ± 2.2
NCR group	478 (93.2)	53.3 ± 0.5
**Total of donors, n (%)**	513	51.2 ± 0.9
CR group	35 (6.8)	44.1 ± 3.6 ^a^
NCR group	478 (93.2)	51.8 ± 0.9
**Total of recipients, n (%)**	513	**CR, n (%)**
Gender		
Male	382 (74.5)	26 (6.8)
Female	131 (25.5)	9 (6.9)
**Transplant indications, n (%)**		
Alcoholic cirrhosis	169 (32.9)	17 (10.1)
Alcoholic cirrhosis plus HCV and/or HBV	54 (10.5)	1 (1.9)
Viral chronic hepatitis	108 (21.1)	10 (9.3)
HCV	91 (17.7)	9 (9.9)
HBV	17 (3.3)	1 (5.9)
Hepatocarcinoma	72 (14.0)	3 (4.2)
Autoimmune diseases	27 (5.3)	2 (7.4)
Fulminant hepatitis	16 (3.1)	0 (0)
Others	67 (13)	2 (3.5)
Virus pre-infected patients *, n (%)		
HCV	155 (30.2)	10 (6.5)
HBV	51 (9.9)	3 (5.9)
CMV	11 (2.1)	0 (0)

n, number of individuals of each group; CMV, cytomegalovirus infection; CR, Chronic rejection; HBV, hepatitis B virus; HCV, hepatitis C virus, NCR, No-chronic rejection, SEM, Standard error of the mean. * Virus pre-infected patients are classified regardless of the type of transplant indication. ^a^ *p* = 0.026; CR and NCR donors mean ages were compared by the two-sided Student *t*-test.

**Table 2:** KIR gene frequencies in healthy donors, total patients and liver recipients with chronic liver rejection.

		Healthy Donors N = 513	Total Patients N = 513		Chronic Rejection N = 35		
KIR Genes	P/A	n (%)	n (%)	P1	n (%)	P2	P3
***iKIRs***							
**2DL1^+^/S1^−^**	+	311 (60.6)	306 (59.6)	0.799	22 (7.2)	0.725	-
	−	202 (39.4)	207 (40.4)		13 (6.3)		
**2DL2**	+	316 (61.6)	296 (57.7)	0.227	27 (9.1)	**0.020 ^b^**	**0.033**
	−	197 (38.4)	217 (42.3)		8 (3.7)		
**2DL3**	+	449 (87.5)	454 (88.5)	0.701	28 (6.2)	0.105	-
	−	64 (12.5)	59 (11.5)		7 (11.9)		
**2DL5**	+	268 (52.2)	285 (55.6)	0.316	21 (7.4)	0.603	-
	−	245 (47.8)	228 (44.4)		14 (6.2)		
**3DL1**	+	490 (95.5)	490 (95.5)	1.000	33 (6.7)	0.665	-
	−	23 (4.5)	23 (4.5)		2 (8.7)		
***aKIRs***							
**2DS1**	+	192 (37.4)	204 (39.8)	0.481	12 (5.9)	0.593	-
	−	321 (62.6)	309 (60.2)		23 (7.4)		
**2DS2**	+	317 (61.8)	292 (56.9)	0.127	27 (9.2)	**0.013 ^c^**	**0.030**
	−	196 (38.2)	221 (43.1)		8 (3.6)		
**2DS3**	+	169 (32.9)	164 (32.0)	0.790	17 (10.4)	**0.038 ^d^**	**0.037**
	−	344 (67.1)	349 (68.0)		18 (5.2)		
**2DS4**	+	491 (95.7)	488 (95.1)	0.766	33 (6.8)	0.685	-
	−	22 (4.3)	25 (4.9)		2 (8.0)		
**2DS5**	+	143 (27.9)	174 (33.9)	**0.043 ^a^**	11 (6.3)	0.854	-
	−	370 (72.1)	339 (66.1)		24 (7.1)		
**3DS1**	+	143 (27.9)	215 (41.9)	0.408	13 (6.0)	0.599	-
	−	370 (72.1)	298 (58.1)		22 (7.4)		

N, total number of individuals in each group; n, number of donors or recipients with each KIR; P/A; presence or absence, aKIR, activating killer cell immunoglobulin-like receptors; iKIR, inhibitory killer cell immunoglobulin-like receptors. All comparations were made by the two-sided Fisher’s exact test. P1, Univariable analysis for comparing healthy donors and patients and the presence and absence for each KIR analyzed P2, univariable analysis for comparing the CR and NCR group, and the presence and absence for each KIR analyzed. P3, Logistic regression multivariable analysis for chronic rejection considered the following variables: age, sex, HCV pre-infection, and KIR in recipients and age, HLA-C allotypes in donors, as well as the KIR gene mismatching between recipient-donor pairs. ^a^ *p* = 0.043; OR = 0.753, 95% CI: 0.577–0.982; ^b^ *p* = 0.020; OR = 0.381, 95% CI: 0.170–0.857; ^c^ *p* = 0.013; OR = 2.713, 95% CI: 1.208–6.094; ^d^ *p* = 0.038; OR = 2.127, 95% CI: 1.066–4.243.

**Table 3:** Match or mismatch of KIR genes between liver recipient-donor pairs and the development of chronic liver rejection.

		Chronic Rejection			
All KIRs	N	n (%)	OR (95% CI)	P1	P2
0 MM	67	3 (4.5)	1.649 (0.491–5.543)	0.603	-
≥1 MM	446	32 (7.2)			
**iKIRs ^a^**					
0 MM	120	3 (2.5)	3.457 (1.040–11.497)	**0.037**	**0.033**
≥1 MM	393	32 (8.1)			
**KIR2DL3**					
0 MM	406	21 (5.2)	2.760 (1.353–5.631)	**0.008**	0.191
1 MM	107	14 (13.1)			
**aKIRs ^b^**					
0 MM	80	4 (5.0)	1.465 (0.503–4.271)	0.632	-
≥1 MM	433	31 (7.2)			

KIR, Killer Immunoglobulin-like Receptors; aKIR; Activating Killer Immunoglobulin-like Receptors; CI, Confidence interval; iKIR, Inhibitory Killer Immunoglobulin-like Receptors; KIR, Killer Immunoglobulin-like Receptors; MM, Mismatch; n, Number of transplant with chronic rejection; N, Total number of transplants; OR, Odds Ratio; ^a^ A total of 5 inhibitory KIR genes (2DL1-3, 2DL5, 3DL1) were considered. ^b^ A total of six activating KIR genes (2DL1-3, 2DL5, 3DL1) were considered. Significant *p* values are marked in bold. P1 values were obtained by logistic regression univariable analysis and P2 values were obtained by multivariable analysis.

**Table 4:** Analysis of HLA-A -B and -C allele mismatches and their influence on the chronic rejection of liver grafts.

		Chronic Rejection			
HLA-A Mismatches	N	n	%	OR (95% CI)	P *
2 MM	328	23	7	1.068 (0.519–2.201)	1.000
1 MM	155	10	6.5	0.910 (0.426–1.944)	1.000
0 MM	27	2	7.4	1.091 (0.248–4.807)	0.707
**HLA-B Mismatches**					
2 MM	385	25	6.5	0.895 (0.406–1.972)	0.837
1 MM	111	9	8.1	1.320 (0.597–2.916)	0.519
0 MM	14	0	0	0.931 (0.909–0.954)	0.614
**HLA-C Mismatches**					
2 MM	341	24	7	1.108 (0.530–2.319)	0.855
1 MM	141	9	6.4	0.907 (0.414–1.987)	1.000
0 MM	31	2	6.5	0.938 (0.214–4.105)	1.000

* All comparations were made by the two-sided Fisher’s exact test. CI, Confidence interval; CR, acute rejection; HLA, Human Leukocyte Antigens; MM, mismatch; n, number of CR patients; N, the total number of transplants; OR, Odds ratio with a confidence interval (CI) of 95%.

**Table 5:** Influence of HLA-C allotypes and genotypes and in chronic rejection liver patients.

**A. Recipient and Donor HLA-C Allotypes and Their Influence in Chronic Rejection Liver**						
	**HLA-C Allotypes**		**Chronic Rejection**			
		**N = 513**	**n**	**%**	**P ^a^**	
**Recipient**	C1^+^	409	31	7.6	0.274	
	C1^−^	104	4	3.8		
	C2^+^	331	22	6.6	0.856	
	C2^−^	182	13	7.1		
**Donor**	C1^+^	429	24	5.6	**0.018 ^b^**	
	C1^−^	84	11	13.1		
	C2^+^	340	26	7.6	0.357	
	C2^−^	173	9	5.2		
**B. Recipient and donor HLA-C genotypes and their influence in chronic rejection liver.**						
			**Chronic rejection**			
	**HLA-C genotypes**	**N = 513**	**n**	**%**	***X^2^***	**P ^a^**
	C1C1	182	13	7.1		0.856
**Recipient**	C1C2	227	18	7.9	0.384	0.384
	C2C2	104	4	3.8		0.274
	C1C1	173	9	5.2		0.357
**Donor**	C1C2	256	15	5.9	**0.043**	0.484
	C2C2	84	11	13.1		**0.018 ^b^**

N, total number of transplants; n, number of CR patients; HLA, Human Leukocyte Antigens. OR, Odds Ratio; CI, Confidence interval; ^a^ Comparations were made by the two-sided Fisher’s exact test. Significant *p* values are marked in bold. ^b^ OR= 0.393, 95% CI: 0.185–0.837; *p* = 0.018.

**Table 6:** Analysis of HLA-C genotypes in recipient-donor pairs and the development of chronic liver rejection.

HLA-C Genotypes Combinations			Chronic Rejection		
Donor	Recipient	N = 513	n	%	P ^a^
	C1C1	65	4	6.2	0.295
C1C1	C1C2	73	5	6.8	
	C2C2	35	0	0	
	C1C1	87	4	4.6	0.806
C1C2	C1C2	118	8	6.8	
	C2C2	51	3	5.9	
	C1C1	30	5	16.7	0.534
C2C2	C1C2	36	5	13.9	
	C2C2	18	1	5.6	

CR, chronic rejection; N, total number of transplants; n, number of CR patients. ^a^ Comparisons were made using a Pearson’s Chi-square test, and significant *p* values are marked in bold.

**Table 7:** KIRs recipients and HLA-I ligand donor combinations and their influence on chronic liver rejection.

			Chronic Rejection				
Recipient KIRs	Donor HLA-I Ligand	N	n	%	OR (95%CI)	P ^a^	P ^b^
***iKIRs***							
**KIR2DL1^+^/S1^−^**	C2^+^	193	17	8.8	2.086 (0.748–5.818)	0.175	-
	C2^–^	113	5	4.4			
**KIR2DL2^+^/S2^+^**	C1^+^	248	19	7.7	0.415 (0.170–1.012)	0.057	-
	C1^–^	48	8	16.7			
**KIR2DL3^+^**	C1^+^	374	18	4.8	0.334 (0.148–0.755)	**0.015**	**0.004**
	C1^–^	76	10	13.2			
**KIR3DL1^+^**	Bw4^+^	387	27	7.0	1.213 (0.487–3.020)	0.826	-
	Bw4^–^	103	6	5.8			
***aKIRs***							
**KIR2DL1^+^/KIR2DS1^+^**	C2^+^	145	8	5.5	0.803 (0.232–2.776)	0.747	-
	C2^–^	59	4	6.8			
	C2^+^	323	25	7.7	1.646 (0.726–3.735)	0.248	-
	C2^–^	165	8	4.8			
**KIR2DS4^+^**							
	C1^+^	406	22	5.4	0.370 (0.172–0.796)	**0.014**	**0.028**
	C1^–^	82	11	13.4			
**KIR2DL3^+^/S4^+^**	C1^+^	353	16	4.5	0.304 (0.132–0.700)	**0.006**	**0.003**
	C1^–^	74	10	13.5			

N, Total number of transplants; n, Number of CR transplants; CI, Confidence interval; OR, Odds Ratio; aKIR, activating killer immunoglobulin-like receptors; HLA, human leukocyte antigen class I; iKIR, inhibitory killer immunoglobulin-like receptors; KIRs, killer immunoglobulin-like receptors. ^a^ Comparison was made by the two-sided Fisher’s exact test. Significant *p* values are marked in bold. ^b^ Significant *p* values obtained by logistic regression multivariable analysis for CR, parameters considering the age and sex of recipient, donor age, HCV, and HLA-C mismatch between donor and recipient.

## Abbreviations

aKIR: activating killer cell immunoglobulin-like receptors; HLA-C1 or C2 ligands: Two HLA-C allotypes; CMV, cytomegalovirus infection; CR: Chronic liver rejection; d: gene present in the donor (dHLA or dKIR); GVHD: graft-versus-host-disease; HBV, hepatitis B virus; HCV, hepatitis C virus; HBsAg: hepatitis B virus surface antigen; HLA-I: Human Leukocyte Antigens; HSCT: Hematopoietic Stem Cell Transplant; iKIR: Inhibitory killer cell immunoglobulin-like receptors; KIR: Killer cell immunoglobulin-like receptors; MM: Mismatching; NCR: No-chronic rejection; NK: Natural Killer cell; r: gene presence in receptor (rHLA or rKIR).
